# Mechanism of FoxO1 in the Metabolic Shift of Fetal Rat Heart

**DOI:** 10.3390/molecules31081275

**Published:** 2026-04-13

**Authors:** William William, Neng Tine Kartinah, Ani Retno Prijanti, Yoga Yuniadi, Prasandhya Astagiri Yusuf, Yow-Pin Lim

**Affiliations:** 1 Doctoral Program in Biomedical Science, Faculty of Medicine, Universitas Indonesia, Jakarta 10430, Indonesia; william@ukrida.ac.id; 2 Department of Physiology, Faculty of Medicine and Health Sciences, Krida Wacana Christian University (UKRIDA), Jakarta 11510, Indonesia; 3 Department of Medical Physiology and Biophysics, Faculty of Medicine, Universitas Indonesia, Jakarta 10430, Indonesia; prasandhya.a.yusuf@ui.ac.id; 4 Department of Biochemistry and Molecular Biology, Faculty of Medicine, Universitas Indonesia, Jakarta 10430, Indonesia; ani.retno@ui.ac.id; 5 Department of Cardiology and Vascular Medicine, Faculty of Medicine, Universitas Indonesia, Jakarta 10430, Indonesia; yoga.yuniadi@ui.ac.id; 6 Medical Technology Indonesian Medical Education and Research Institute (IMERI), Faculty of Medicine, Universitas Indonesia, Jakarta 10430, Indonesia; 7 Department of Pathology and Laboratory Medicine, The Alpert Medical School of Brown University, Providence, RI 02903, USA; yow-pin_lim_md@brown.edu

**Keywords:** fetal heart, FoxO1, hypoxia, metabolic shift

## Abstract

Cardiovascular diseases remain a leading cause of morbidity and mortality worldwide, underscoring the need to better understand cardiovascular physiology. A key aspect involves identifying regulatory molecules that govern metabolic shifts. Forkhead box protein O1 (FoxO1) has emerged as a potential regulator; however, its role and underlying mechanisms remain unclear. This study investigated FoxO1 in metabolic adaptation using Wistar rats divided into age groups (fetal, postnatal day 1, postnatal day 7, adult) and treatment groups (control, hypoxia, FoxO1 inhibitor, combination). Hypoxia (12–14% O_2_) and FoxO1 inhibitor (AS1842856, 10 mg/kgBW/day) were administered accordingly. Parameters assessed included hypoxia inducible factor 1 α (HIF-1α), FoxO1 mRNA and protein, glucose transporter type 1 (GLUT1), glucose transporter type 4 (GLUT4), cluster of differentiation 36 (CD36), hexokinase, pyruvate dehydrogenase kinase isoform 4 (PDK4), phosphoenolpyruvate carboxykinase (PEPCK), lactic acid, malonyl-CoA, carnitine palmitoyltransferase 1 (CPT1), citrate synthase, cytochrome c, and adenosine triphosphate (ATP). ATP production increased with age, associated with higher FoxO1 expression and metabolic shifts. Hypoxia in fetal hearts reduced HIF-1α and FoxO1. FoxO1 inhibition elevated glycolytic and oxidative markers. In conclusion, FoxO1 regulates glycolysis and lipid metabolism, offering insights into cardiac adaptation to hypoxia and potential therapeutic strategies.

## 1. Introduction

Cardiovascular diseases (CVDs) consist of diseases of the heart and blood vessels [1], and are the leading cause of death and morbidity worldwide [2]. In 2022, an estimated 19.8 million people died from CVDs, which was around 32% of all deaths globally [3]. In developed countries such as the United States in 2020, the incidence of CVDs was around 48% for 20-year-olds, and the percentage increases with age [4]. In Indonesia, the prevalence of heart disease in 2023 was 0.85%, and it is one of the main causes of death in Indonesia, and the largest burden of health costs [5].

Cardiovascular diseases (CVDs), particularly coronary artery disease, are characterized by myocardial hypoxia, a condition in which oxygen supply to heart tissue is insufficient at the cellular level [4]. This hypoxic state can impair myocardial contractility, reduce adenosine triphosphate (ATP) production, and lead to tissue damage [6]. Interestingly, the fetal heart demonstrates greater tolerance to hypoxia than the adult heart [7]. This adaptive capacity is attributed to three main factors: the presence of fetal hemoglobin (HbF), which enhances oxygen transport [8]; the brain-sparing mechanism, which prioritizes oxygen delivery to vital organs [9]; and a distinct metabolic profile in which the fetal heart relies primarily on glucose rather than fatty acids for energy [10]. As glucose metabolism consumes less oxygen than fatty acid oxidation, the fetal heart is inherently more resistant to hypoxic stress. In postnatal life, a metabolic shift occurs, favoring fatty acid utilization, which increases oxygen demand and reduces hypoxia tolerance [11].

The metabolic shift is regulated by several protein molecules, including heart and neural crest derivatives expressed 1 (HAND1) [12], myeloid ectotrophic viral integration site 1 (MEIS1) [13], and p38γ/δ [14]. HAND1 is highly expressed in the fetal heart, associated with hypoxia via hypoxia inducible factor 1 α (HIF-1α), and plays a role in suppressing lipid metabolism [12]. MEIS1 is highly expressed in the fetal heart, where it promotes glucose metabolism and suppresses lipid metabolism, although it is not directly associated with hypoxia [13]. Expression of p38γ/δ increases two weeks after birth, unrelated to hypoxia, and contributes to enhanced lipid metabolism [14]. Each of these metabolic shift regulators has limitations: HAND1 is not associated with acute hypoxia [12], while MEIS1 and p38γ/δ show no clear relationship with hypoxic conditions [13,14].

Forkhead box protein O1 (FoxO1) is a member of the forkhead (FOX) transcription factor family, which includes FoxO1, FoxO3a, FoxO4, and FoxO6 [15]. Among these, FoxO1 plays a central role in regulating cellular metabolism, including heart metabolic processes [16,17]. It is expressed during both fetal and adult stages, with sustained postnatal expression, making it relevant to adult heart physiology [18]. This molecule also contributes to the hypoxic response via hypoxia inducible factor 1 α (HIF-1α) [19], and is involved in both glucose and lipid metabolism [17]. A selective FoxO1 inhibitor (AS1842856) is already available [20], enabling targeted modulation and offering a promising therapeutic avenue. Given its multifaceted functions, FoxO1 is considered a strong candidate for regulating metabolic shifts. However, its role in metabolic shift and fetal heart metabolism has yet to be demonstrated. Elucidating the function of FoxO1 in metabolic shift may provide a scientific basis for developing therapies that mimic fetal metabolism to counteract hypoxia in the adult heart. Wistar rats were chosen as the experimental model because they are widely used in hypoxia induction studies [21], and the cellular and transcriptomic profiles of their cardiovascular system closely resemble those of humans [22], thereby enhancing the translational relevance of this study. This study aims to investigate the role of FoxO1 in metabolic shift and its contribution to fetal heart metabolism, providing a foundation for more effective interventions in hypoxia-associated cardiovascular conditions.

## 2. Results and Discussion

### 2.1. Metabolic Transition from Fetal Rat Hearts to Adult Rat Hearts

**Figure 1:**
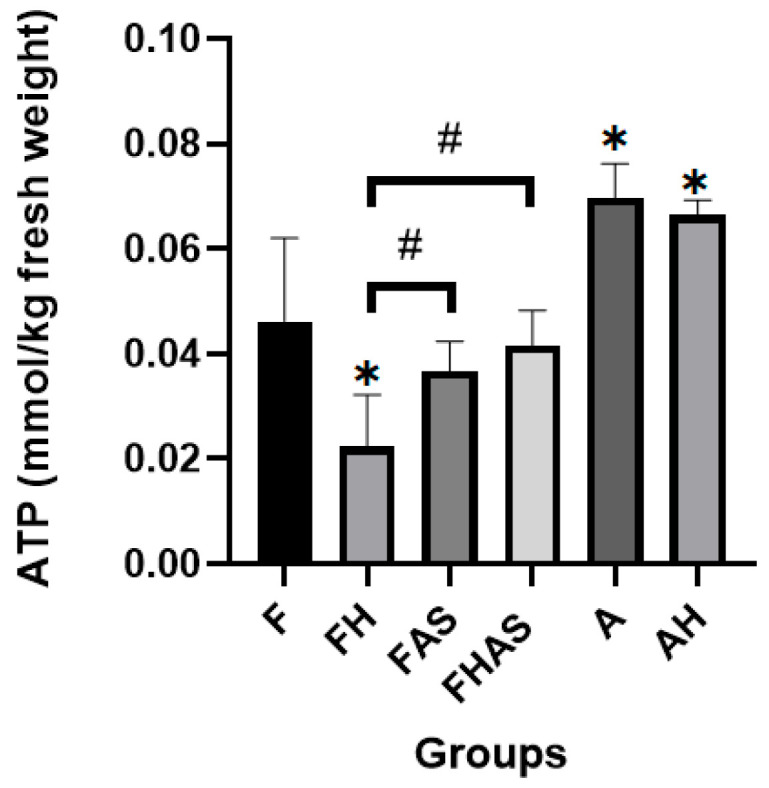
Adenosine triphosphate (ATP) concentration in rats’ heart tissue following hypoxia and AS1842856 treatment (*n* = 4). Data is presented as mean ± SD. F: fetal rat, FH: fetal rat + hypoxia, FAS: fetal rat + AS1842856, FHAS: fetal rat + hypoxia + AS1842856, A: adult rat, AH: adult rat + hypoxia. * *p* < 0.05 compared to F, # *p* < 0.05 compared to other groups.

Adenosine triphosphate (ATP) serves as the primary energy source for cells, and the heart exhibits a particularly high demand for ATP to sustain its function as a pump [23]. ATP can be generated through glucose or fatty acid metabolism, depending on physiological conditions and developmental stage [23]. In this study, adult hearts exhibited higher ATP levels than fetal hearts (0.070 ± 0.007 mmol/kg fresh weight vs. 0.046 ± 0.016 mmol/kg fresh weight; MD = 0.024, 95% CI (0.010, 0.036); *p* = 0.002) (Figure 1). This difference reflects a metabolic shift from fetal to adult heart energy utilization, in which fetal hearts predominantly rely on glucose metabolism, resulting in lower ATP production, whereas adult hearts primarily utilize fatty acids, leading to higher ATP levels [11].

Hypoxia significantly reduced ATP levels in fetal rats. The hypoxic fetal group showed markedly lower ATP concentrations compared to controls (0.022 ± 0.010 mmol/kg fresh weight vs. 0.046 ± 0.016 mmol/kg fresh weight; MD = −0.024, 95% CI (−0.037, −0.010); *p* = 0.001). In adult rats, hypoxia decreased ATP levels, with the hypoxic group exhibiting lower concentrations than the non-hypoxic group (0.066 ± 0.003 mmol/kg fresh weight vs. 0.069 ± 0.007 mmol/kg fresh weight; MD = −0.003, 95% CI (−0.016, 0.099); *p* = 0.6); however, this difference was not statistically significant (Figure 1). These findings indicate that hypoxia reduces ATP in both fetal and adult rats, although the effect is significant only in the fetal group. The decrease in ATP under hypoxic conditions may be explained by impaired mitochondrial oxidative phosphorylation, which could limit the cellular energy supply and compromise metabolic homeostasis [24]. This observation is consistent with the findings of Wang D et al. [25], who reported that five hours of hypoxia significantly reduced ATP levels in neonatal rat heart myocytes. Notably, ATP levels subsequently recovered and even exceeded baseline during the reoxygenation phase. In our study, administration of AS1842856, a selective FoxO1 inhibitor, was associated with elevated ATP levels under hypoxic conditions (Figure 1), and adequate ATP is essential for maintaining cardiac pump function during hypoxia [26]. The specificity of AS1842856 and its effects in cardiac tissue remain incompletely characterized. Therefore, these findings should be interpreted with caution, and further studies are needed to clarify whether the observed increase in ATP is directly mediated by FoxO1 modulation or by other molecular targets.

### 2.2. FoxO1 mRNA Expression Aligns with the Metabolic Transition from Fetal Rat Hearts to Adult Rat Hearts

**Figure 2:**
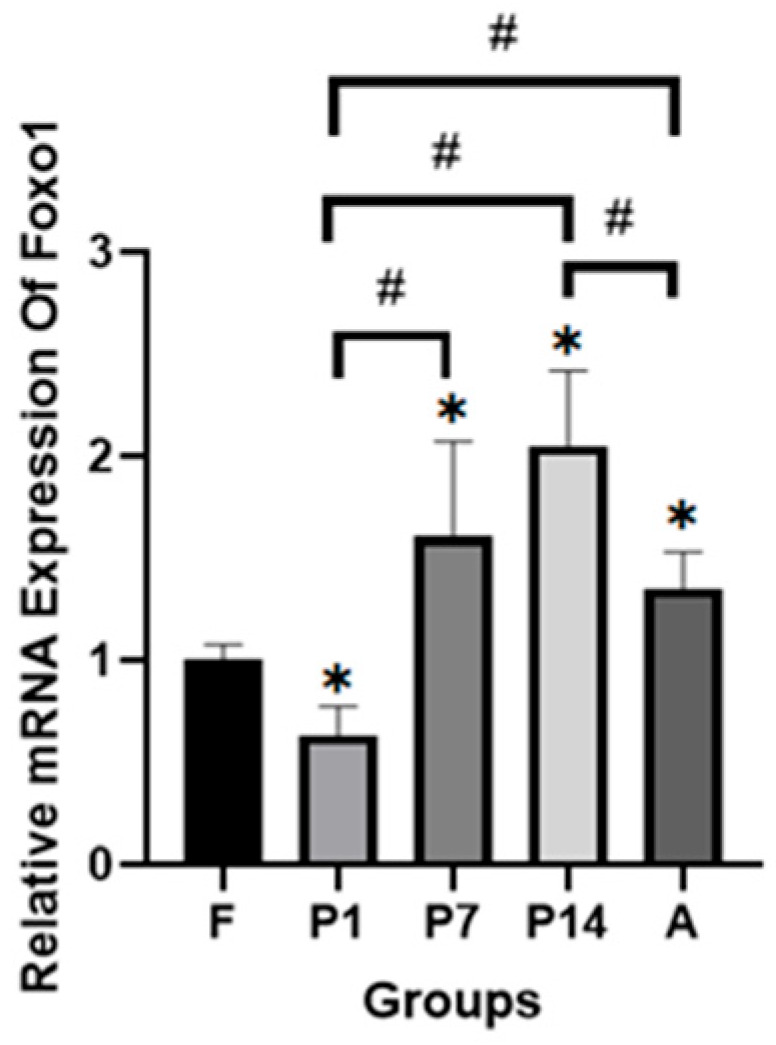
Forkhead box protein O1 (FoxO1) mRNA expression with age in rats’ heart tissue (*n* = 5). Data is presented as mean ± SD. F: fetal rat, P1: 1-day-old rat, P7: 7-day-old rat, P14: 14-day-old rat, A: Adult rat. * *p* < 0.05 compared to F, # *p* < 0.05 compared to other groups.

FoxO1 mRNA expression increased progressively with age, peaking at postnatal day 14 (P14). Although expression in adult hearts (A) was lower than at postnatal day 7 (P7) and 14 (P14), it remained higher than in the fetal group (F). This pattern indicates that metabolic reprogramming continues with age (Figure 2). The observed trend in FoxO1 mRNA expression suggests its involvement in the metabolic shift from a fetal to an adult heart phenotype. This result aligns with the findings of Santamans AM et al. [14], who identified p38γ and p38δ as key regulators of postnatal metabolic reprogramming, with protein expression increasing with age and peaking at postnatal day 14.

Our findings suggest that FoxO1 may contribute to the postnatal metabolic shift. However, this transition represents a complex process involving multiple regulatory molecules. Previous studies have also implicated HAND1, MEIS1, p38γ, and p38δ in metabolic reprogramming [12,13,14]. HAND1 suppresses lipid metabolism in fetal hearts by inhibiting fatty acid entry into cells via fatty acid binding protein (FABP), and by blocking mitochondrial fatty acid transport through malonyl-CoA inhibition [12]. MEIS1 enhances glucose metabolism by upregulating aldolase and enolase enzymes, while concurrently inhibiting lipid metabolism through suppression of fatty acid translocase (FAT) in fetal hearts [13]. p38γ and p38δ promote postnatal lipid metabolism and inhibit glucose utilization by downregulating glucose transporter type 4 (GLUT4) and pyruvate kinase, and increasing β-oxidation activity [14]. Based on our findings and prior reports, we hypothesize that HAND1 and MEIS1 regulate the prenatal metabolic shift, while p38γ, p38δ, and FoxO1 potentially govern the postnatal transition.

### 2.3. Hypoxia Induced Downregulation of HIF-1α Leads to Reduced FoxO1 Protein Expression in Fetal Rat Hearts

**Figure 3:**
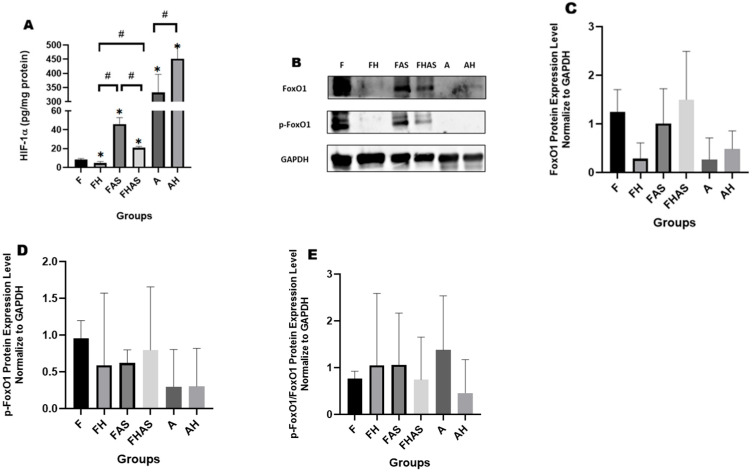
Effect of hypoxia and FoxO1 inhibition on HIF-1α, FoxO1, and p-FoxO1 protein expression in rats’ heart tissue. (**A**) HIF-1α levels (*n* = 4) presented as mean ± SD; * *p* < 0.05 compared to F, # *p* < 0.05 compared to other groups. (**B**) Representative Western blot of FoxO1 and p-FoxO1 protein expression in rats’ heart tissue, with glyceraldehyde-3-phosphate dehydrogenase (GAPDH) as loading control. (**C**–**E**) Densitometric quantification of FoxO1, p-FoxO1, p-FoxO1/FoxO1 band intensities from three independent experiments (*n* = 3); normalized to GAPDH, presented as mean ± SD. F: fetal rat, FH: fetal rat + hypoxia, FAS: fetal rat + AS1842856, FHAS: fetal rat + hypoxia + AS1842856, A: adult rat, AH: adult rat + hypoxia. Full scan (uncropped) original Western blot images are provided in Figures S2–S10.

In our study, hypoxia reduced HIF-1α protein levels in fetal hearts (4.57 ± 1.78 pg/mg protein vs. 8.50 ± 0.99 pg/mg protein; log10 MD = −0.29, 95% CI (−0.41, −0.16); GMR = 0.52, 95% CI (0.39, 0.69); *p* = 0.0001). The opposite occurred in adult hearts following hypoxic induction (451.68 ± 45.06 mg protein vs. 332.23 ± 64.29 mg protein; log10 MD = 0.13, 95% CI (0.01, 0.26); GMR = 1.35, 95% CI (1.02, 1.82); *p* = 0.032) (Figure 3A). This reduction contrasts with the findings of Bae S et al. [27], who reported increased HIF-1α expression in Sprague-Dawley fetal hearts exposed to hypoxia during gestational days 15–20. The discrepancy may be attributed to differences in oxygen concentration: our study used 12–14% oxygen, whereas Bae S et al. [27] used 10%. Subject variation may also contribute, as we used Wistar rats, whereas Bae S et al. [27] used Sprague Dawley rats, which are known to exhibit superior adaptive responses to hypoxia [28]. Despite this, Wistar rats remain the preferred model for cardiovascular disease research due to their cellular diversity and genetic expression patterns that more closely resemble the human cardiovascular system [22]. The observed decrease in HIF-1α levels in fetal rat hearts under hypoxia may be explained by several mechanisms, including chronic hypoxia induced negative feedback that enhances HIF-1α degradation via prolyl hydroxylase domain (PHD) and Von Hippel–Lindau (VHL) proteins [29,30], and translational disruption through mTOR/MAPK pathway modulation [31]. In addition to canonical feedback mechanisms, the reduction in HIF-1α may also represent a maladaptive stress response, in which chronic hypoxia exceeds the fetal heart’s compensatory capacity [32,33,34]. These mechanisms may collectively suggest a transition from physiological compensation to pathological alteration, potentially indicating that the hypoxic exposure in this model exceeds the fetal heart’s tolerance threshold.

Hypoxia not only reduced HIF-1α levels in fetal hearts but also tended to reduce FoxO1 expression (Figure 3B), suggesting a possible correlation between hypoxic exposure and FoxO1 regulation. The relationship between HIF-1α and FoxO1 may be bidirectional, as indicated by the finding that in fetal hearts treated with the FoxO1 inhibitor AS1842856, HIF-1α levels increased (Figure 3A). Densitometric quantification of FoxO1 protein levels (*n* = 3) was consistent with this trend (Figure 3C), although the small sample size precludes a definitive statistical conclusion. Similarly, while total FoxO1 levels were assessed, analysis of phosphorylated FoxO1 (p-FoxO1) showed a decreasing trend in the AS1842856-treated (FAS) group (Figure 3D); nonetheless, as with the total protein data, these observations remain preliminary and should be interpreted with caution due to the limited number of replicates. Consequently, the p-FoxO1/FoxO1 ratio showed an increasing tendency (Figure 3E), which may reflect enhanced cytoplasmic sequestration and reduced nuclear activity of FoxO1 [35]. Although a limitation of this study is the absence of direct measurement of AS1842856 levels in maternal or fetal tissues to confirm drug delivery, the observed reduction in fetal body weight (Figure S1), together with the phosphorylation shift, provides preliminary functional evidence that AS1842856 likely reached the fetus and that FoxO1 transcriptional activity could have been suppressed under our experimental conditions.

Nevertheless, the reproducible pattern across experiments suggests that hypoxia tends to decrease FoxO1 abundance, while FoxO1 inhibition partially restores its expression. Although this observation is consistent with a potential reciprocal relationship between HIF-1α and FoxO1, the limited sample size precludes definitive conclusions. Despite this limitation, these findings support the possibility that hypoxia-driven changes in HIF-1α may be associated with alterations in FoxO1 expression; further investigation is needed to establish a direct causal link. The link between hypoxia and FoxO1 appears to be mediated by the protein kinase B-mechanistic target of rapamycin complex 1 (AKT–mTORC1) and AMP-activated protein kinase (AMPK) signaling pathways, which coordinate cellular responses to metabolic stress [36,37]. The hypoxia induced downregulation of FoxO1 in fetal hearts aligns with the findings of Chen CJ et al. [19], who reported decreased FoxO1 mRNA and protein expression in H9c2 cells following 24 h of hypoxic exposure. The similarity between fetal heart tissue and H9c2 cells suggests a conserved regulatory mechanism in which hypoxia suppresses FoxO1 expression. This consistency between in vitro and in vivo results strengthens the hypothesis that hypoxia triggers metabolic adaptation involving FoxO1 downregulation, thereby altering gene expression and cellular function in the fetal heart.

### 2.4. FoxO1 as a Putative Negative Regulator of Glucose Metabolism in Fetal Rat Hearts

**Figure 4:**
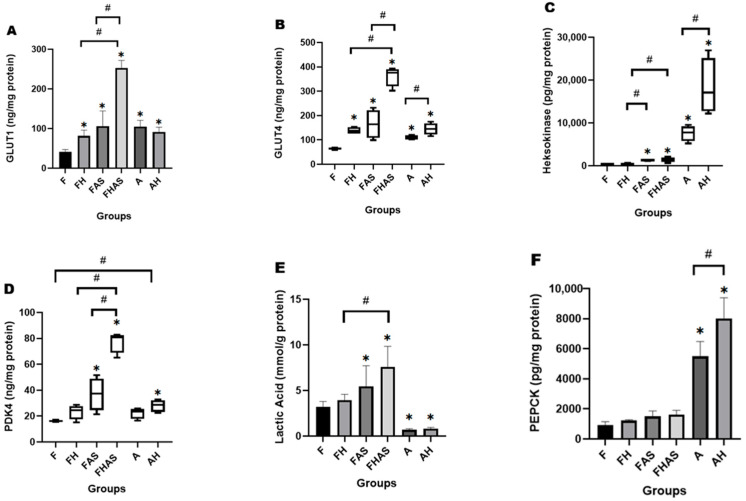
Effect of hypoxia and FoxO1 inhibitor on glucose metabolism in rats’ heart tissue (*n* = 4). (**A**) glucose transporter type 1 (GLUT1) level, (**B**) glucose transporter type 4 (GLUT4) level, (**C**) Hexokinase level, (**D**) pyruvate dehydrogenase kinase isoform 4 (PDK4) level, (**E**) phosphoenolpyruvate carboxykinase (PEPCK) level, (**F**) Lactic acid level. GLUT1, Lactic Acid, and PEPCK are presented as mean ± SD; GLUT4, Hexokinase, and PDK4 are presented as median and interquartile range (IQR) (box-and-whisker plots), the horizontal line within the box represents the median, while whiskers indicate the minimum and maximum values. * *p* < 0.05 compared to F, # *p* < 0.05 compared to other groups. F: fetal rat, FH: fetal rat + hypoxia, FAS: fetal rat + AS1842856, FHAS: fetal rat + hypoxia + AS1842856, A: adult rat, AH: adult rat + hypoxia.

FoxO1 plays a critical role in metabolic regulation. To investigate its function in fetal heart metabolism, we inhibited FoxO1 activity using the selective inhibitor AS1842856. In our study, FoxO1 inhibition was associated with increased expression of glucose transporter type 1 (GLUT1) and glucose transporter type 4 (GLUT4), as shown in Figure 4A,B, suggesting that FoxO1 may suppress glucose uptake. The fetal with FoxO1 inhibitor FAS group exhibited a significant increase in GLUT1 expression compared to the fetal (F) group (105.78 ± 38.58 ng/mg protein vs. 41.02 ± 6.03 mg protein; MD = 64.75, 95% CI (34.40, 95.09); *p* = 0.0001). Similarly, GLUT4 expression was higher in the FAS group than in the F group (164.09 (99.27–231.94) ng/ mg protein vs. 64.88 (59.35–67.66) ng/ mg protein, U = 0.000, *p* = 0.021). These findings align with the study by Ying F et al. [38], which similarly demonstrated that prostaglandin receptor knockout, resulting in reduced FoxO1 levels, led to increased expression of GLUT1 and GLUT4.

Glucose entering the cell undergoes glycolysis, with hexokinase serving as the first enzyme in this pathway [39]. In our study, hexokinase levels in the FAS group were significantly higher than those in the F group (1350.29 (1160.67–1424.99) pg/mg protein vs. 363.88 (228.02–490.38) pg/mg protein, U = 0.000, *p* = 0.021), indicating that FoxO1 may suppress hexokinase expression (Figure 4C). These findings suggest that FoxO1 may inhibit glucose uptake in fetal rat cardiomyocytes and potentially impair glycolysis. This result aligns with the findings of Battiprolu PK et al. [40], who reported that mice fed a high-fat diet exhibited increased FoxO1 expression and reduced hexokinase activity. Enhanced glucose uptake and glycolysis were also observed in the hypoxia-treated group (FH), with a synergistic effect in the group receiving both treatments (hypoxia + FoxO1 inhibitor (FHAS)) (Figure 4A–C). The observed increase in glycolysis may contribute to cardio protection under hypoxic conditions, as supported by previous studies showing that elevated glycolytic flux during ischemia–reperfusion injury enhances energy availability and reduces reactive oxygen species (ROS) production [41]. Therefore, our results suggest that FoxO1 inhibition promotes functional glucose utilization and may offer broader therapeutic benefits in the management of myocardial hypoxia.

As glycolysis progresses, pyruvate is formed and funneled into mitochondrial metabolism through conversion to acetyl-CoA by pyruvate dehydrogenase. This enzyme is inhibited by pyruvate dehydrogenase kinase isoform 4 (PDK4) [39]. PDK4 is a downstream target of FoxO1, and a previous study has demonstrated that FoxO1 binds directly to the PDK4 promoter to enhance its transcription [42]. This FoxO1–PDK4 axis plays an important role in the heart, where it contributes to the regulation of metabolic adaptation and energy homeostasis [35]. In our study, FoxO1 inhibition was associated with increased PDK4 levels in the FAS group compared to the F group (37.45 (21.50–51.62) ng/mg protein vs. 16.19 (15.41–17.01) ng/mg protein, U = 0.000, *p* = 0.029), suggesting a possible regulatory role of FoxO1 in PDK4 expression (Figure 4D). The modulation of PDK4 expression observed here provides an additional line of preliminary functional evidence that FoxO1 transcriptional activity could have been suppressed under our experimental conditions. This result contrasts with the findings of Yan D et al. [35], who reported that FoxO1 inhibition reduced both mRNA and protein levels of PDK4 in the hearts of adult diabetic Sprague-Dawley rats. The discrepancy may be explained by the activation of an alternative regulatory axis involving peroxisome proliferator-activated receptor delta (PPARδ) [43]. During late gestation (E15–E20), the fetal heart undergoes a critical metabolic shift from carbohydrate reliance toward enhanced fatty acid utilization [44]. Under these conditions, FoxO1 inhibition relieves its antagonistic constraint on nuclear receptor signaling, thereby permitting PPARδ to drive transcriptional programs [45], such as PDK4 induction [43]. Another possible explanation lies in the fetal dependence on maternal nutrient supply [46]. When FoxO1 is inhibited, fatty acid utilization in the maternal heart may decrease, increasing substrate availability for the fetal heart. Elevated fatty acid availability has been reported to stimulate PDK4 expression [47], thereby providing a plausible mechanism for the observed increase in PDK4 levels. Although our data suggest that FoxO1 inhibition may increase PDK4 expression through alternative regulatory pathways such as PPARδ activation or altered maternal–fetal substrate availability, we did not obtain direct mechanistic evidence to confirm these interactions. The absence of direct experimental validation represents a limitation of our study. Future investigations employing targeted assays (e.g., chromatin immunoprecipitation for FoxO1/PPARδ binding or metabolic flux analysis under controlled substrate conditions) will be required to substantiate these proposed mechanisms.

In our study, increased PDK4 levels in the FAS group may have contributed to reduced pyruvate oxidation to acetyl-CoA, accompanied by elevated lactate production. Lactate levels in the FAS group were significantly higher than in the F group (5.45 ± 2.26 mmol/g protein vs. 3.23 ± 0.57 mmol/g protein; log10 MD = 0.13, 95% CI (0.04, 0.37); GMR = 1.35, 95% CI (1.10, 2.34); *p* = 0.017) (Figure 4E), a finding that contrasts with Udawant S et al. [48], who reported that FoxO1 inhibition in human glioblastoma cells (U87MG) reduced lactate dehydrogenase A (LDHA) expression and lactate levels. This apparent discrepancy does not indicate a contradiction; rather, it underscores that the role of FoxO1 is highly context-dependent, varying across tissue types and physiological states. In fetal hearts, FoxO1 appears to maintain metabolic balance by suppressing glycolytic flux and lactate production, whereas in cancer cells, FoxO1 sustains LDHA expression as part of a proliferative strategy [48,49]. FoxO1 inhibition did not notably affect phosphoenolpyruvate carboxykinase (PEPCK) expression; although PEPCK levels in the FAS group were elevated, the values did not differ significantly from those observed in the F group (1501.23 ± 362.34 pg/mg protein vs. 931.03 ± 223.81 pg/mg protein; MD = 570.20; 95% CI (−492.02, 1632.42); *p* = 0.274) (Figure 4F). This modest change may have occurred because PEPCK expression in the heart is primarily stimulated by increased cardiac workload, and PEPCK contributes to both gluconeogenesis and cardiac hypertrophy [50].

Our findings suggest that FoxO1 may function as a negative regulator of glucose metabolism in fetal rat hearts. It appears to suppress GLUT1 and GLUT4 expression, limit glycolysis through hexokinase downregulation, and modulate PDK4 expression and lactate production. These functional observations are complemented by structural insights from RCSB-PDB and AlphaFold models, which highlight key domains of FoxO1 relevant to DNA binding and transcriptional regulation (Figure S11). Such perspectives support the potential mechanistic role of FoxO1 in fetal heart metabolism and overall metabolic control, although further studies are required to confirm these interactions. Importantly, the possibility that postnatal inhibition of glucose metabolism contributes to cardiac development is consistent with prior reports linking sustained glucose dominance in the postnatal heart to cardiomyopathy [12].

### 2.5. FoxO1 as a Potential Regulator of Fatty Acid Utilization in Fetal Rat Hearts

**Figure 5:**
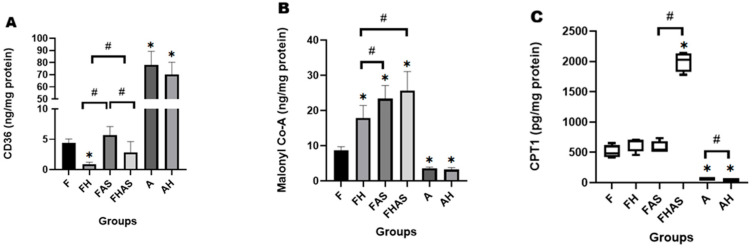
Effect of hypoxia and FoxO1 inhibitor on lipid metabolism in rats’ heart tissue (*n* = 4). (**A**) cluster of differentiation 36 (CD36) level, (**B**) Malonyl Co-A level, (**C**) carnitine palmitoyltransferase 1 (CPT1) level. CD36 and Malonyl Co-A are presented as mean ± SD; CPT1 is presented as median and interquartile range (IQR) (box-and-whisker plots), the horizontal line within the box represents the median, while whiskers indicate the minimum and maximum values. * *p* < 0.05 compared to F, # *p* < 0.05 compared to other groups. F: fetal rat, FH: fetal rat + hypoxia, FAS: fetal rat + AS1842856, FHAS: fetal rat + hypoxia + AS1842856, A: adult rat, AH: adult rat + hypoxia.

FoxO1, while primarily known for its role in glucose metabolism, also regulates lipid metabolism. Similarly to the approach used for glucose metabolism, the role of FoxO1 in lipid metabolism was assessed by inhibiting its function using the selective FoxO1 inhibitor (AS1842856). In our study, FoxO1 inhibition did not significantly alter the expression of lipid transporters. Figure 5A shows that cluster of differentiation 36 (CD36) levels in the FAS group were higher than in the F group (5.66 ± 1.42 ng/mg protein vs. 4.40 ± 0.65 ng/mg protein; log10 MD = 0.27, 95% CI: (−0.41, 0.95); GMR = 1.86, 95% CI (0.39, 8.91); *p* = 0.414). However, this difference was not statistically significant. To ensure that this non-significant result was not due to an insufficient sample size, a post hoc power analysis was conducted. The analysis revealed that despite the small sample size (*n* = 4), this study maintained adequate statistical power to detect large effect sizes. In the case of CD36, the observed lack of significance likely reflects a true minimal biological effect of FoxO1 inhibition on CD36 expression under these specific conditions, rather than a lack of statistical power. This finding is consistent with the study by Gopal K et al. [51], who reported that FoxO1 inhibition did not affect CD36 mRNA or protein expression in the hearts of type 2 diabetic mice. In contrast, Ying F et al. [38] demonstrated that EP4 receptor knockout reduced FoxO1 expression, leading to a decrease in cardiac CD36 expression. This discrepancy may be due to a direct regulatory pathway between EP4 and CD36, and differences in the methods used to assess protein function (inhibitor vs. knockout). This distinction of methods represents a limitation of this study. However, further investigation is needed to clarify these mechanisms.

In our study, malonyl-CoA concentrations were significantly higher in the FAS group compared to the F group (23.34 ± 3.76 ng/mg protein vs. 8.75 ± 0.95 ng/mg protein; log10 MD = 0.42, 95% CI (0.31–0.52); GMR = 2.63, 95% CI (2.04–3.31); *p* = 0.0001), suggesting that FoxO1 may contribute to the regulation of malonyl-CoA levels (Figure 5B). This finding is consistent with a study by Bastie CC et al. [52], who demonstrated that FoxO1 suppresses malonyl-CoA levels, thereby enhancing fatty acid oxidation in mouse skeletal muscle. Malonyl-CoA is known to inhibit carnitine palmitoyl transferase 1 (CPT1), thereby limiting fatty acid entry into mitochondria [39]. However, our data showed that FoxO1 inhibition did not appear to significantly affect CPT1 levels. Although CPT1 concentrations in the FAS group were numerically higher than in the F group (529.19 (522.38–731.37) pg/mg protein vs. 481 (413.79–652.14) pg/mg protein, U = 3.00, *p* = 0.149) (Figure 5C), this difference was not statistically significant. The stability of CPT1 levels following FoxO1 inhibition may not necessarily reflect its enzymatic activity. This notion is supported by Onay-Besikci A et al. [53], who reported increased CPT1 activity and enhanced fatty acid oxidation in newborn rabbits, without changes in CPT1 mRNA expression. This phenomenon is attributed to reduced malonyl-CoA levels as a modulatory factor [53].

These findings suggest that FoxO1 may act as a potential regulator of lipid metabolism, particularly through its association with reduced malonyl-CoA levels. Although FoxO1 inhibition was associated with a numerical change in CD36 expression, this difference was not statistically significant, and its potential impact on CPT1 remains to be clarified, particularly regarding enzymatic activity in the fetal heart. Postnatal regulation of fatty acid metabolism is critical for cardiac maturation, consistent with evidence that supplementation with fatty acids in human pluripotent stem cell-derived cardiomyocytes (hPSC-CMs) induced cardiomyocyte hypertrophy and significantly enhanced contractile strength [54]. These functional findings and structural insights (Figure S11) suggest that FoxO1 mediated enhancement of lipid metabolism may play an important role in cardiomyocyte maturation, potentially contributing to the development of functionally competent cells with effective contractile capacity.

### 2.6. FoxO1 May Influence Energy Metabolism by Modulating Citrate Synthase and Cytochrome C in Fetal Rat Hearts

**Figure 6:**
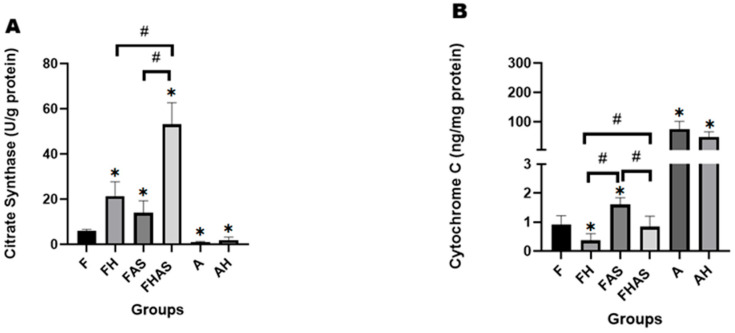
Effect of hypoxia and FoxO1 inhibitor on mitochondrial function in rats’ heart tissue (*n* = 4). (**A**) Citrate synthase activity level. (**B**) Cytochrome C level. Data is presented as mean ± SD. * *p* < 0.05 compared to F, # *p* < 0.05 compared to other groups. F: fetal rat, FH: fetal rat + hypoxia, FAS: fetal rat + AS1842856, FHAS: fetal rat + hypoxia + AS1842856, A: adult rat, AH: adult Rat + hypoxia.

The metabolic pathways of glucose and fatty acids intersect through the generation of acetyl-CoA, a central substrate for the citric acid cycle and mitochondrial oxidative phosphorylation [39]. In our study, citrate synthase activity was significantly higher in the FAS group compared to the F group (13.98 ± 5.31 U/g protein vs. 6.07 ± 0.57 U/g protein; log10 MD = 0.34; 95% CI (0.09, 0.58); GMR = 2.19, 95% CI (1.23, 3.80); *p* = 0.009), suggesting that FoxO1 may influence mitochondrial activity at the level of the citric acid cycle (Figure 6A). This result differs from the findings of Battiprolu PK et al. [40], who reported that a high-fat diet in adult mice significantly increased citrate synthase activity, with a stronger response in wild-type mice compared to cardiomyocyte-specific FoxO1 knockout mice. The discrepancy may be explained by differences in the experimental subjects. In the adult heart, FoxO1 plays an adaptive role in response to metabolic load, including enhanced fatty acid oxidation and activation of mitochondrial enzymes such as citrate synthase [40]. Conversely, in the fetal heart, FoxO1 appears to disrupt normal cardiac metabolism and suppress mitochondrial activity [55], resulting in a distinct response compared to the adult heart with respect to citrate synthase levels.

Analysis of cytochrome c revealed a comparable pattern, with the FAS group showing a significant increase compared to the F group (1.60 ± 0.23 ng/mg protein vs. 0.92 ± 0.29 ng/mg protein; log10 MD = 0.25, 95% CI (0.0033, 0.5083); GMR = 1.78, 95% CI (1.01, 3.22); *p* = 0.047) (Figure 6B). This finding contrasts with the study by Wang D et al. [56], who reported that treatment with AS1842856 reduced cytochrome c expression in human proximal tubular kidney cells. This discrepancy may be explained by differences in developmental stage and tissue type. Our study utilized fetal cardiac tissue undergoing active development, in which FoxO1 functions as a regulator of metabolic programming. Inhibition of FoxO1 reduced lipid metabolism, thereby lowering metabolic load and mitochondrial stress [57]. Under these conditions, cytochrome c expression may increase as part of the recovery of oxidative function [58]. In contrast, Wang D et al. [56] investigated adult human proximal tubular kidney cells, where FoxO1 plays a greater role in oxidative stress responses and apoptosis regulation. In this context, FoxO1 inhibition reduced cytochrome c expression as part of decreased mitochondrial activity and protection against cellular damage [56].

In our study, FoxO1 inhibition was associated with changes in mitochondrial metabolism, reflected by reduced citrate synthase activity and lower cytochrome c expression. These alterations may represent a mechanism to regulate energy balance during development. Such regulation has the potential to alter the heart’s metabolic trajectory and support postnatal adaptation. A decrease in citrate synthase activity is known to promote the metabolic shift of the fetal heart toward fatty acid utilization, resembling the metabolic pattern of the adult heart [59]. On the other hand, impaired function of cytochrome c oxidase has been associated with cellular aging and reduced myocardial contractility [60], while mitochondrial component deficiency, such as COX6A2, leads to metabolic dysfunction, oxidative stress, disturbances in calcium homeostasis, and diminished contractile performance [61]. Overall, these functional findings and structural insights (Figure S11) highlight the importance of mitochondrial regulation in developmental programming and age-related cardiac remodeling, as well as the need for further understanding of the molecular mechanisms that maintain mitochondrial efficiency across the lifespan.

### 2.7. Proposed Mechanism of FoxO1 as a Regulator of Metabolic Shift in Fetal Rat Heart

**Figure 7:**
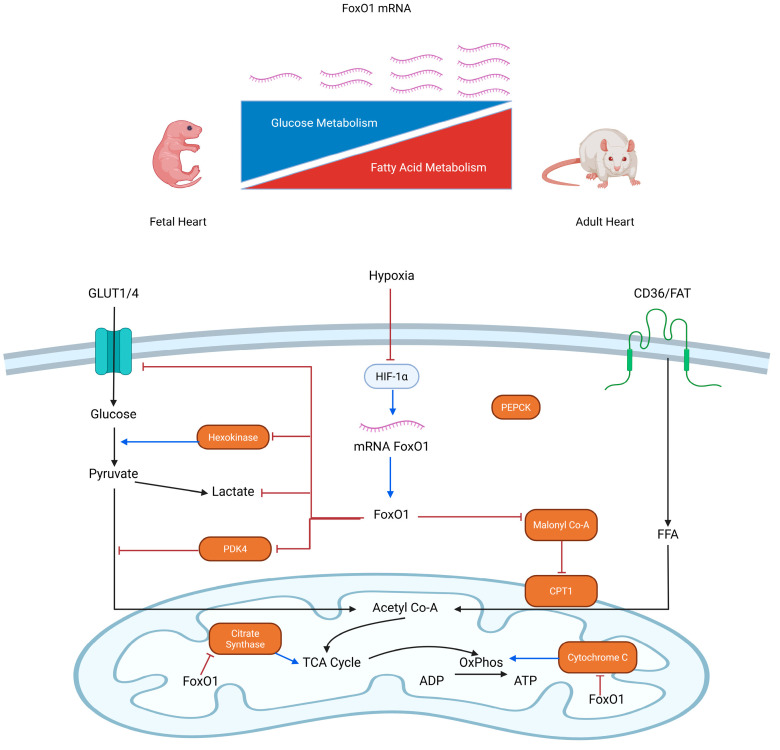
Schematic Representation of FoxO1 Mechanism of Metabolic Shift in Fetal Rat Heart. Abbreviations: adenosine diphosphate (ADP); adenosine triphosphate (ATP); carnitine palmitoyltransferase 1 (CPT1); fatty acid translocase (FAT); free fatty acid (FFA); forkhead box O1 (FoxO1); glucose transporter (GLUT); hypoxia-inducible factor 1 alpha (HIF-1α); oxidative phosphorylation (OxPhos); phosphoenolpyruvate carboxykinase (PEPCK); pyruvate dehydrogenase kinase isoform 4 (PDK4); tricarboxylic acid cycle (TCA cycle). Created in BioRender. William, W. (2026) https://BioRender.com/dczp0av, accessed on 9 April 2026.

FoxO1 mRNA expression increased during fetal cardiac maturation in rats, suggesting a possible role in regulating the shift in energy substrate utilization from glucose to fatty acids. This upregulation was accompanied by decreased expression of GLUT1, GLUT4, and hexokinase, which may contribute to reduced glycolytic activity. Although reducing PDK4 could theoretically enhance pyruvate dehydrogenase (PDH) complex activity, the overall regulatory trend appeared to favor lipid metabolism. In addition, the decline in malonyl-CoA levels may have relieved CPT1 inhibition, thereby facilitating fatty acid entry into mitochondria. Despite this, citrate synthase activity and cytochrome c expression also decreased, consistent with reduced mitochondrial activity (Figure 7). Altogether, these molecular adaptations support the possibility that FoxO1 contributes to the metabolic transition of the fetal rat heart from glucose reliance toward fatty acid oxidation, while potentially limiting glycolytic flux and modulating mitochondrial oxidative capacity.

### 2.8. Summary of Principal Findings, Comparison with the Literature, and Study Limitations

Our study suggests that FoxO1 may act as a regulator of metabolic adaptation in the fetal rat heart, potentially limiting glucose uptake (GLUT1/GLUT4) and glycolysis (hexokinase), while influencing lipid metabolism through malonyl-CoA regulation. Hypoxia reduced both HIF-1α and FoxO1 expression in fetal hearts, whereas FoxO1 inhibition was associated with enhanced glycolytic flux and ATP availability, suggesting a possible therapeutic avenue for hypoxia-induced energy deficiency. These findings highlight the possibility of reciprocal regulation between HIF-1α and FoxO1, and suggest that their combined activity may contribute to metabolic responses under oxygen stress.

Compared with the published literature, our results both converge with and diverge from prior reports. For instance, Bae S et al. [27] reported increased HIF-1α in Sprague-Dawley fetal hearts under hypoxia, whereas we observed a reduction in Wistar rats, likely reflecting differences in oxygen concentration and strain-specific adaptive capacity. Similarly, FoxO1 inhibition increased PDK4 expression in our fetal model, whereas in diabetic adult hearts, it reduced PDK4 expression [35]. These discrepancies highlight the context-dependent role of FoxO1, which varies across developmental stages, tissue types, and metabolic environments.

Despite these insights, several limitations must be acknowledged. First, the small sample size, while statistically powered for large effects, limits generalizability. Second, both total FoxO1 and phosphorylated FoxO1 (p-FoxO1) were assessed from only three independent experiments (*n* = 3), which does not meet the minimum sample size for robust statistical inference. These findings should therefore be interpreted with caution; nevertheless, the trends observed provide valuable preliminary insights into FoxO1 regulation. Third, mechanistic pathways such as FoxO1–PPARδ–PDK4 interactions were inferred but not directly validated, underscoring the need for targeted assays. Fourth, the absence of direct measurement of AS1842856 levels in maternal or fetal tissues precludes definitive confirmation of drug delivery; future pharmacokinetic studies will be essential to establish drug distribution and exposure profiles. Finally, the use of a selective FoxO1 inhibitor (AS1842856) rather than genetic knockout models represents a methodological limitation. While chemical inhibition provides translational relevance, it may not fully recapitulate the complete loss-of-function phenotype of FoxO1. Inhibitors can exert off-target effects and do not fully eliminate FoxO1 activity, thereby limiting mechanistic resolution. Nevertheless, this approach offers a practical proof of concept for therapeutic modulation. Future studies employing genetic knockout or knockdown strategies will be required to validate and extend these findings.

## 3. Materials and Methods

### 3.1. Laboratory Animals

Wistar rats were obtained from PT Bio Farma (Persero), Bandung, Indonesia. The animals were healthy, immunocompetent, non-genetically modified, and had not undergone any prior experimental procedures. All rats underwent a one-week acclimatization period. All animals were housed and treated at the Animal Research Facilities (ARF), Indonesia Medical Education and Research Institute (IMERI), Faculty of Medicine, Universitas Indonesia.

The inclusion criteria for this study, established a priori, involved three categories of experimental units: (1) a non-mating male group (8–10 weeks old, 240–320 g), (2) a mating parental group comprising males (8–10 weeks old, 240–320 g) and females (8–10 weeks old, 182–218 g); (3) the resulting offspring generation, restricted to males, including 20-day-old fetuses and neonates at 1, 7, and 14 days of age. All animals examined for experimental parameters in this study were male. Male sex in late-gestation fetuses, postnatal, and adult rats was identified by a greater anogenital distance and slight pigmentation [62]. Exclusion criteria included animals exhibiting signs of illness, anatomical abnormalities, or significant distress. Throughout this study, no animals or data points were excluded from the analysis, ensuring that the final *n* for each group remained as initially planned. Animals were housed in a quiet room at 25 ± 2 °C with 65 ± 10% humidity, 3–4 rats per cage, and provided with food and water ad libitum.

### 3.2. Experimental Design

This study was an experimental study and had received ethical approval from the Ethical Comitte of the Faculty of Medicine, Universitas Indonesia-Cipto Mangunkusumo Hospital (KET-1318/UN2.F1/ETIK/PPM.00.02/2023).

The research subjects were divided into two groups based on age and treatment. The age grouping was designed to examine the expression pattern of FoxO1 mRNA across developmental stages. Rats were divided into five groups: fetal (20 days, F), postnatal day 1 (P1), postnatal day 7 (P7), postnatal day 14 (P14), and adult (8–10 weeks, A). The minimum sample size for each group was calculated using Federer’s formula, yielding 5 rats per group (total 25 rats). Each individual rat was considered an experimental unit, and fetal rats (F) served as the control group for baseline comparisons for the developmental stage.

The treatment grouping was designed to evaluate the relationship between hypoxia and FoxO1 inhibition with heart metabolism by measuring parameters such as HIF-1α, FoxO1 protein expression, GLUT1, GLUT4, CD36, hexokinase, PDK4, PEPCK, lactic acid, malonyl-CoA, CPT1, citrate synthase, cytochrome C, and ATP. Hypoxia induction was performed using a custom-built hypoxia chamber (50 × 50 × 50 cm), calibrated to maintain oxygen levels at 12–14%. The choice of 12–14% O_2_ concentration was based on both references in the literature and preliminary experimental observations. Hypoxia induction in pregnant rats can be achieved with oxygen levels of 10% or less, as well as levels between 12 and 14% [63]. In our unpublished pilot experiments, exposure to 10% O_2_ resulted in high maternal mortality, with three out of four pregnant dams dying during the intervention. The single surviving dam produced only four pups, compared to the normal litter size of 8–12 in rats. In contrast, the use of 12–14% O_2_ allowed for maternal survival and yielded viable litters, thereby providing a physiologically relevant model of moderate hypoxia without excessive lethality. For these reasons, we selected 12–14% O_2_ as the experimental condition.

The chamber was equipped with continuous monitoring of O_2_ concentration to ensure reproducibility of the hypoxic environment. Normobaric hypoxia was applied continuously throughout the treatment period. FoxO1 inhibition was achieved with daily oral administration of AS1842856 (10 mg/kg BW) during the designated treatment period. Fetal rats were obtained by mating male and female rats, and pregnancy was confirmed by the presence of spermatozoa in vaginal swabs. Gestational age was designated as day 0 upon detection of spermatozoa. Rats were divided into six groups: fetal rats aged 20 days (F); fetal rats with hypoxia induction (12–14% O_2_, FH) at 15–20 weeks of gestation; fetal rats treated with FoxO1 inhibitor (AS1842856, 10 mg/kgBW/day, FAS) at 15–20 weeks of gestation; fetal rats with combined hypoxia and FoxO1 inhibitor (FHAS) at 15–20 weeks of gestation; adult rats aged 8–10 weeks (A); and adult rats subjected to hypoxia induction (12–14% O_2_, AH) for 5 days. The minimum sample size for each group was calculated using Federer’s formula, yielding 4 rats per group (total 24 rats). Each individual rat was considered an experimental unit, and fetal rats (F) and adult rats (A) served as the control groups for baseline comparisons of developmental stage and treatment effects.

Animals were randomly assigned to experimental groups, and all procedures were carried out under standardized conditions to minimize variability. At the conclusion of this study, rats were anesthetized with an intramuscular injection of ketamine (87 mg/kg BW) and xylazine (13 mg/kg BW) prior to cardiac tissue collection. To ensure objectivity, outcome assessments were conducted using a single-blinded approach, with the laboratory analyst remaining unaware of treatment allocation until all parameters had been evaluated. Potential confounders such as substrate availability and cardiac workload were controlled by maintaining uniform feeding schedules, providing identical chow diets, and housing animals under consistent environmental conditions. All treatments and measurements were performed at the same time of day to reduce fluctuations in metabolic responses.

Rat hearts harvested under anesthesia were immediately rinsed with ice-cold 0.9% NaCl to remove residual blood. To ensure maximal molecular stability, samples were processed according to their designated applications: tissues for protein and metabolic analysis were placed in pre-chilled tubes and stored at −80 °C, while those for gene expression were submerged in RNAlater (Thermo Fisher Scientific, #AM7024, Waltham, MA, USA). These were kept at 4 °C overnight for penetration before the supernatant was removed, and tissues were transferred to −80 °C. Subsequently, heart tissues for protein and metabolic analysis were homogenized in ice-cold 0.9% NaCl using a mechanical homogenizer (Tokyo Rikakikai Co., Ltd., Tokyo, Japan). The homogenates were centrifuged at 12,000× *g* for 15 min, and the supernatants were collected for further analysis. Total protein concentration was determined by the Bradford assay (Thermo Fisher Scientific #J61522.K2, Waltham, MA, USA), using bovine serum albumin (BSA) as a standard for normalization.

#### 3.2.1. Gene Expression Analysis

- RNA Isolation

Total RNA was extracted using the Quick-RNA™ MiniPrep Plus kit (Zymo Research #R1057, Irvine, CA, USA) according to the manufacturer’s protocol. Lysates were filtered with a spin-away filter and centrifuged (12,000 *g*, 30 min). The supernatant was mixed with ethanol (1:1) and loaded onto a Zymo-Spin III CG column. After an initial wash with RNA wash buffer, DNase I treatment (5 μL in 75 μL DNA digestion buffer) was performed and incubated for 15 min at room temperature. Washing continued with RNA Prep Buffer followed by two washes with RNA wash buffer, and RNA was eluted with 50 μL of DNase/RNase-free water. RNA concentration and purity were measured using a Varioskan™ LUX multimode microplate reader (Thermo Fisher Scientific #VL0000D0 with a μDrop™ Plate #N12391, Waltham, MA, USA). RNA concentration was calculated as A260 × 40 μg/mL × dilution factor, and purity was determined using the A260/A280 ratio (1.65–2).

- qRT-PCR

mRNA expression was analyzed using SYBR Green–based qRT-PCR on an Applied Biosystems 7500 Real-Time PCR system, Thermo Fisher Scientific, Waltham, MA, USA. Reactions were prepared as a master mix containing 2× SensiFAST™ SYBR^®^ No-ROX One-Step Mix (#BIO-72005), forward and reverse primers (0.8 μL each, 10 μM), RNase inhibitor (0.4 μL), reverse transcriptase (0.2 μL), RNA template (100 ng), and nuclease-free water to a final volume of 20 μL. Primers for the target gene FoxO1 were forward (CGCCAAACACCAGTCTAAATTC) and reverse (CTGTCCCTGAAGTGTCTGCATAG), while glyceraldehyde-3-phosphate dehydrogenase (GAPDH) served as the internal control with forward (GAAGGTCGGTGTGAACGGAT) and reverse (AACTTGCCGTGGGTAGAGTC) primers. The thermal protocol included reverse transcription at 45 °C for 10 min, pre-denaturation at 95 °C for 2 min, followed by cycles of denaturation at 95 °C for 5 s, annealing at 60 °C for 10 s, and extension at 72 °C for 5 s. Relative expression analysis was performed using the Livak (ΔΔCt) method.

#### 3.2.2. Bradford Assay

Total protein concentration was determined using the Bradford method with a bovine serum albumin (BSA) standard solution (1 mg/mL) over the concentration range 0–1 mg/mL. A 10 μL sample or standard was mixed with 200 μL of Bradford reagent (Thermo Fisher Scientific #J61522.K2) and incubated for 5 min at room temperature. Absorbance was measured at 595 nm using a Varioskan™ LUX multimode microplate reader (Thermo Fisher Scientific #VL0000D0, Waltham, MA, USA).

#### 3.2.3. Enzyme-Linked Immunosorbent Assay (ELISA) Assay

- Hypoxia-Inducible Factor 1 Alpha (HIF-1α)

HIF-1α levels were determined using the Rat HIF-1α ELISA kit (Elabscience, #E-EL-R0513, Wuhan, China) according to the manufacturer’s protocol. A total of 100 μL of sample or standard was added to each well and incubated at 37 °C for 90 min. After the liquid was removed, 100 μL of biotinylated antibody was added and incubated for 60 min. The plate was washed three times with 350 μL of wash buffer, then 100 μL of horseradish peroxidase (HRP)-conjugate was added and incubated for 30 min. Following five washes 90 μL of substrate reagent was added; after 15 min of incubation, 50 μL of stop solution was added to stop the reaction. The absorbance at 450 nm was measured using a Varioskan™ LUX multimode microplate reader (Thermo Fisher Scientific #VL0000D0, Waltham, MA, USA).

- Glucose Transporter 1 (GLUT1) and Glucose Transporter 4 (GLUT4)

GLUT1 and GLUT4 levels were determined using ELISA kits (BT Lab #E1087Ra and BT Lab, #E0499Ra, Jiaxing, China) according to the manufacturer’s protocol. Samples (40 μL) were mixed with specific antibodies (10 μL of rat solute carrier family 5 member 1 (SLC5A1) or solute carrier family 2 member 4 (SLC2A4), while standards consisted of 50 μLof standard solution and 50 μL of HRP. Plates were incubated at 37 °C for 60 min and washed five times with 300 μL of wash buffer. Substrate solutions A and B (50 μL each) were added, the reaction was incubated for 10 min, and then terminated with 50 μL of the stop solution. The absorbance at 450 nm was measured using a Varioskan™ LUX multimode microplate reader (Thermo Fisher Scientific #VL0000D0, Waltham, MA, USA).

- Hexokinase

Hexokinase levels were determined using the Rat HK (Hexokinase) ELISA kit (FineTest, #ER1038, Wuhan, China) according to the manufacturer’s protocol. Samples and standards (100 μL) were added to the well plate and incubated at 37 °C for 90 min, followed by two washes with 350 μL of wash buffer. Afterward, 100 μL of biotin-labeled antibody was added and incubated for 60 min, followed by three washes. The streptavidin–biotin complex (SABC) working solution (100 μL) was subsequently added, incubated for 30 min, and then subjected to five washes. Following the washing step, 90 μL of 3, 3′, 5, 5′-tetramethylbenzidine (TMB) substrate was added. The mixture was incubated for 30 min, and the reaction was terminated by adding 50 μL of stop solution. The absorbance at 450 nm was measured using a Varioskan™ LUX multimode microplate reader (Thermo Fisher Scientific #VL0000D0, Waltham, MA, USA).

- Pyruvate Dehydrogenase Kinase 4 (PDK4)

PDK4 levels were determined using the Rat Pyruvate Dehydrogenase Kinase Isoform 4 ELISA kit (MyBiosource, #MBS1602984, San Diego, CA, USA) according to the manufacturer’s protocol. Samples (40 μL) were mixed with 10 μL of biotinylated antibody and 50 μL of streptavidin, while standards consisted of 50 μL of standard solution and 30 μL of HRP solution. Plates were incubated at 37 °C for 60 min and washed five times with 300 μL of wash buffer. Substrate solutions A and B (50 μL each) were added, and the mixture was incubated for 10 min. The reaction was then terminated with 50 μL of stop solution. The absorbance at 450 nm was measured using a Varioskan™ LUX multimode microplate reader (Thermo Fisher Scientific #VL0000D0, Waltham, MA, USA).

- Phosphoenolpyruvate Carboxykinase (PEPCK)

PEPCK levels were determined using the Rat PEPCK (Phosphoenolpyruvate Carboxykinase) ELISA kit (FineTest, #ER1251, Wuhan, China) according to the manufacturer’s protocol. Samples or standards (100 μL) were added to the well plate and incubated at 37 °C for 90 min, followed by two washes with 350 μL of wash buffer. A volume of 100 μL of biotin-labeled antibody was then added and incubated for 60 min, with three subsequent washes. SABC solution (100 μL) was added, incubated for 30 min, and then subjected to five washes. After washing, 90 μL of TMB substrate was introduced and incubated for 10 min. The reaction was then terminated with 50 μL of stop solution. The absorbance at 450 nm was measured using a Varioskan™ LUX multimode microplate reader (Thermo Fisher Scientific #VL0000D0, Waltham, MA, USA)

- CD36/Fatty Acid Translocase (FAT)

CD36/FAT levels were determined using the Rat GP4/CD36 ELISA kit (FineTest, #ER0013, Wuhan, China) according to the manufacturer’s protocol. Samples or standards (100 μL) were added to the well plate and incubated at 37 °C for 90 min, followed by two washes with 350 μL of wash buffer. Subsequently, 100 μL of biotinylated antibody was added and incubated for 60 min, then washed three times. SABC solution (100 μL) was added and incubated for 30 min, followed by five washes. TMB substrate was added, followed by a 30 min incubation, and the reaction was terminated with 50 μL of the stop solution. The absorbance at 450 nm was measured using a Varioskan™ LUX multimode microplate reader (Thermo Fisher Scientific #VL0000D0, Waltham, MA, USA).

- Malonyl-CoA

Malonyl-CoA levels were determined using the Rat Malonyl Coenzyme A ELISA kit (CUSABIO, #CSB-E12893r, Houston, TX, USA) according to the manufacturer’s protocol. Samples or standards (50 μL) were mixed with 50 μL of HRP solution and incubated at 37 °C for 40 min. Four washes with 200 μL of wash buffer were performed prior to the additon of 90 μL TMB substrate, which was incubated for 20 min. The reaction was then terminated with 50 μL of the stop solution. The absorbance at 450 nm was measured using a Varioskan™ LUX multimode microplate reader (Thermo Fisher Scientific #VL0000D0, Waltham, MA, USA).

- Carnitine Palmitoyltransferase 1 (CPT1)

CPT1 levels were determined using the Rat Carnitine Palmitoyltransferase 1B ELISA kit (MyBioSource, #MBS102328, San Diego, CA, USA) according to the manufacturer’s protocol. Samples or standards (50 μL) were added to the well plate, along with 100 μL of HRP conjugate, and incubated at 37 °C for 60 min. After four washes with 350 μL of wash buffer, 50 μL of chromogen A and 50 μL of chromogen B were added, and the mixture was incubated for 15 min. The reaction was then terminated with 50 μL of the stop solution. The absorbance at 450 nm was measured using a Varioskan™ LUX multimode microplate reader (Thermo Fisher Scientific #VL0000D0, Waltham, MA, USA).

- Cytochrome c

Cytochrome c levels were determined using the Rat Cyt-C ELISA kit (Elabscience, #E-EL-R0006, Wuhan, China) according to the manufacturer’s protocol. Samples or standards (100 μL) were added to the wells and incubated at 37 °C for 90 min. After removal of the liquid, 100 μL of biotinylated antibody was added and incubated for 60 min, followed by three washes with 350 μL of wash buffer. Subsequently, 100 μL of HRP-conjugate working solution was added and incubated for 30 min, then washed five times. The substrate reagent (90 μL) was added, following a 20 min incubation, the reaction was then terminated with 50 μL of the stop solution. Absorbance was measured at 450 nm using a Varioskan™ LUX multimode microplate reader (Thermo Fisher Scientific #VL0000D0, Waltham, MA, USA).

#### 3.2.4. Western Blot Analysis

Protein separation was performed using SDS-PAGE with Bio-Rad TGX Stain-Free™ FastCast™ Acrylamide, Hercules, CA, USA. Samples were mixed with sample buffer, heated, and loaded into the gel together with protein markers, then electrophoresed at a steady applied voltage of 120 V for 90 min. Separated proteins were transferred to polyvinylidene difluoride (PVDF) membranes (0.45 µm pore size) using a semi-dry transfer system at 40 V and 40 mA for 120 min at 4 °C. Membranes were blocked with 5% BSA in phosphate-buffered saline with Tween (PBST)/tris-buffered saline with tween (TBST) for one hour, followed by overnight incubation at room temperature with primary antibodies: FoxO1 Rabbit mAb (Cell Signaling, Danvers, MA, USA, #C29H4, 1:500), p-FoxO1 Cell Signalling, Danvers, MA, USA, Rabbit mAb (Ser256) #9461, and GAPDH Mouse mAb (Cell Signaling, Danvers, MA, USA, #97166, 1:2000). After washing, membranes were incubated for one hour with HRP-conjugated secondary antibodies: mouse anti-rabbit IgG-HRP, Santa Cruz, CA, USA, #sc-2357 (1:1000 for FoxO1 and p-FoxO1) and goat anti-mouse IgG-HRP Elabscience, Wuhan, China, #E-AB-1001 (1:10,000 for GAPDH). Signal detection was performed using chemiluminescent substrate and visualized with a UVITEC Cambridge instrument, Cambridge, UK. The immunoblot images were horizontally flipped during figure preparation to align the sample order with the narrative flow of this study. All band identifications were verified against the original molecular weight markers. Densitometric quantification of band intensities was performed using ImageJ software version 1.54g, Bethesda, MD, USA from three independent experiments (*n* = 3), with FoxO1 signals normalized to GAPDH. The images presented are cropped from the original full-length membranes, and the unedited, full-scan raw data are provided in the Supplementary Materials.

#### 3.2.5. Colorimetric Assay

- Lactic Acid

Lactic acid levels were determined using the L-Lactic Acid Colorimetric Assay Kit (Elabscience, #E-BC-K044-S, Wuhan, China), according to the manufacturer’s protocol. A total of 20 μL of ddH_2_O was added to an Eppendorf tube as a blank, 20 μL of the standard solution was used as a control, and 20 μL of the sample was used for analysis. Each tube was then supplemented with 1000 μL of the enzyme working solution and 200 μL of the chromogenic agent, mixed thoroughly, and incubated at 37 °C for 10 min. Subsequently, 2000 μL of the stop solution was added. Absorbance was measured using a cuvette at 530 nm with a Thermo Scientific GENESYS 10S Series spectrophotometer, Thermo Fisher Scientific, Waltham, MA, USA.

- Citrate Synthase

Citrate synthase activity was analyzed using the Citrate Synthase (CS) Activity Assay Kit (Elabscience, #E-BC-K178-M, Wuhan, China), according to the manufacturer’s protocol. Each well was supplemented with 125 μL of buffer solution, 30 μL of substrate, and 20 μL of chromogenic agent, mixed for 3 s, and incubated at 37 °C for 3 min. Subsequently, 10 μL of sample or standard was added, and mixed again for 3 s, and the initial absorbance (OD_1_) was recorded at 412 nm. Incubation was continued for 8 min at the same temperature, after which the second absorbance (OD_2_) was measured at 412 nm using a Varioskan™ LUX multimode microplate reader (Thermo Fisher Scientific #VL0000D0, Waltham, MA, USA).

- ATP

ATP concentration was analyzed using the ATP Colorimetric Assay Kit (Elabscience, #E-BC-K157-S, Wuhan, China), according to the manufacturer’s protocol. Standard solution (30 μL) was mixed with 330 μL of control working solution, while samples were mixed with 330 μL of detection working solution and incubated at 37 °C for 30 min. Following incubation, 50 μL of protein precipitator was added, and the mixture was centrifuged at 1000 *g* for 5 min to obtain 300 μL of supernatant. The supernatant was combined with 500 μL chromogenic agent working solution, mixed for 5 s, and left at room temperature for 2 min. Subsequently, 500 μL of the stop solution was added, mixed for 5 s, and left for 5 min. Absorbance was measured at 636 nm using a cuvette with a Thermo Fisher Scientific GENESYS 10S Series spectrophotometer, Waltham, MA, USA.

### 3.3. Statistical Analysis

Statistical analysis was performed using IBM SPSS 25 Statistics software, Armonk, NY, USA. Data normality was first tested with the Shapiro–Wilk test. If the data were not normally distributed and group variances were not homogeneous, data transformation was applied. When the data were normally distributed and had homogeneous variances, the analysis continued with a one-way ANOVA. Conversely, if the data were non-normal and variances were not homogeneous, the non-parametric Kruskal–Wallis test was used. All statistical tests were conducted with a significance threshold of *p* < 0.05. Furthermore, a post hoc power analysis was conducted using G*Power 3.1.9.7, Heinrich-Heine-Universität Düsseldorf, Düsseldorf, Germany, to evaluate the statistical power (1 − β) of this study, given the observed effect sizes and a sample size of *n* = 4 per group.

## 4. Conclusions

This study is the first to report the role of FoxO1 in the cardiac metabolic shift in rats, suggesting FoxO1 as a critical regulator of metabolic adaptation by redirecting energy utilization from glucose metabolism in the fetal heart to fatty acid metabolism in the adult heart. In parallel, we observed that hypoxia reduced HIF-1α levels in fetal Wistar rat hearts, a finding that contrasts with the commonly reported stabilization of HIF-1α during hypoxia. This divergence, likely influenced by oxygen concentration and strain differences, underscores the importance of model selection in interpreting hypoxic responses. Together, the downregulation of HIF-1α and FoxO1 highlights a maladaptive transition in fetal hearts, providing valuable insight into the mechanisms underlying cardiac adaptation to hypoxia and pointing to potential therapeutic targets to mitigate myocardial damage associated with coronary heart disease. Further investigations using adult rats with coronary heart disease are warranted to determine whether FoxO1 inhibition can attenuate the deleterious effects of hypoxia on cardiac muscle.

## Data Availability

The data presented in this study are available on request from the corresponding author.

## Supplementary Materials

The following supporting information can be downloaded at https://www.mdpi.com/article/10.3390/molecules31081275/s1. Figure S1: Effect of hypoxia and FoxO1 inhibitor on fetal rats’ heart and body weight. Figure S2: FoxO1 Western blot for Figure 3B. Figure S3: p-FoxO1 Western blot for Figure 3B. Figure S4: GAPDH Western blot for Figure 3B. Figure S5: FoxO1 Western blot (second dataset). Figure S6: p-FoxO1 Western blot (second dataset). Figure S7: GAPDH Western blot (second dataset). Figure S8: FoxO1 Western blot (third dataset). Figure S9: p-FoxO1 Western blot (third dataset). Figure S10: GAPDH Western blot (third dataset). Figure S11: Structural model of FoxO1 DNA-binding domain interacting with a target DNA element.

## Abbreviations

The following abbreviations are used in this manuscript:

AKT–mTORC1	protein kinase B-mechanistic target of rapamycin complex 1
AMPK	AMP-activated protein kinase
ATP	adenosine triphosphate
BSA	bovine serum albumin
CA	California
CD36	cluster of differentiation 36
CPT1	carnitine palmitoyltransferase 1
CVDs	cardiovascular diseases
FABP	fatty acid binding protein
FAT	fatty acid translocase
FOX	forkhead
FoxO1	forkhead box protein O1
GAPDH	glyceraldehyde-3-phosphate dehydrogenase.
GLUT1	glucose transporter type 1
GLUT4	glucose transporter type 4
HAND1	heart and neural crest derivatives expressed 1
HRP	horseradish peroxidase
HbF	fetal hemoglobin
HIF-1α	hypoxia inducible factor 1 α
hPSC-CMs	human pluripotent stem cell-derived cardiomyocytes
LDHA	lactate dehydrogenase a
MA	Massachusetts
MD	Maryland
MEIS1	myeloid ecotropic viral integration site 1
NY	New York
ON	Ontario
PBST/TBST	phosphate-buffered saline with tween/tris-buffered saline with tween
PDH	pyruvate dehydrogenase
PDK4	pyruvate dehydrogenase kinase isoform 4
PEPCK	phosphoenolpyruvate carboxykinase
PHD	prolyl hydroxylase domain
PPARδ	peroxisome proliferator-activated receptor delta
PVDF	polyvinylidene difluoride
ROS	reactive oxygen species
SABC	streptavidin–biotin complex
SLC2A4	solute carrier family 2 member 4
SLC5A1	solute carrier family 5 member 1
RI	rhode island
TMB	3,3′,5,5′-tetramethylbenzidine
TX	Texas
UK	United Kingdom
USA	United States of America
VHL	von hippel–lindau
WI	Wisconsin
