# Supplementary material for: Mechanism of FoxO1 in the Metabolic Shift of Fetal Rat Heart
Source: Molecules. 2026 Apr 13;31(8):1275. doi: 10.3390/molecules31081275 (PMC13118310; doi:10.3390/molecules31081275)
Supplement: Supplementary file 1 [file molecules-31-01275-s001.zip › molecules-4149754-supplementary.pdf]

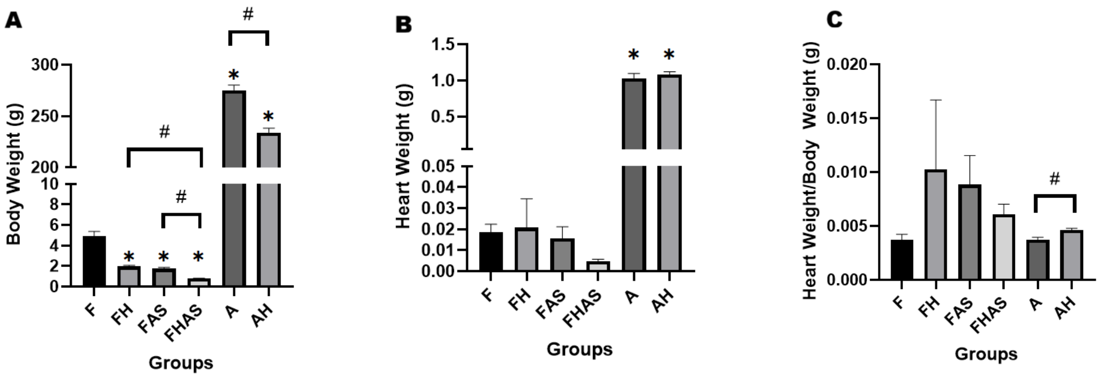

Figure S1: Effect of hypoxia and FoxO1 inhibitor on fetal rats' heart and body weight (n=4). A) Body Weight, B) Heart Weight, C) Heart Weight/Body Weight. Data is presented as mean ± SD. F: fetal rat, P1: 1-day-old rat, P7: 7-day-old rat, P14: 14-day-old rat, A: Adult rat. *p<0.05 compared to F, #p<0.05 compared to other groups.

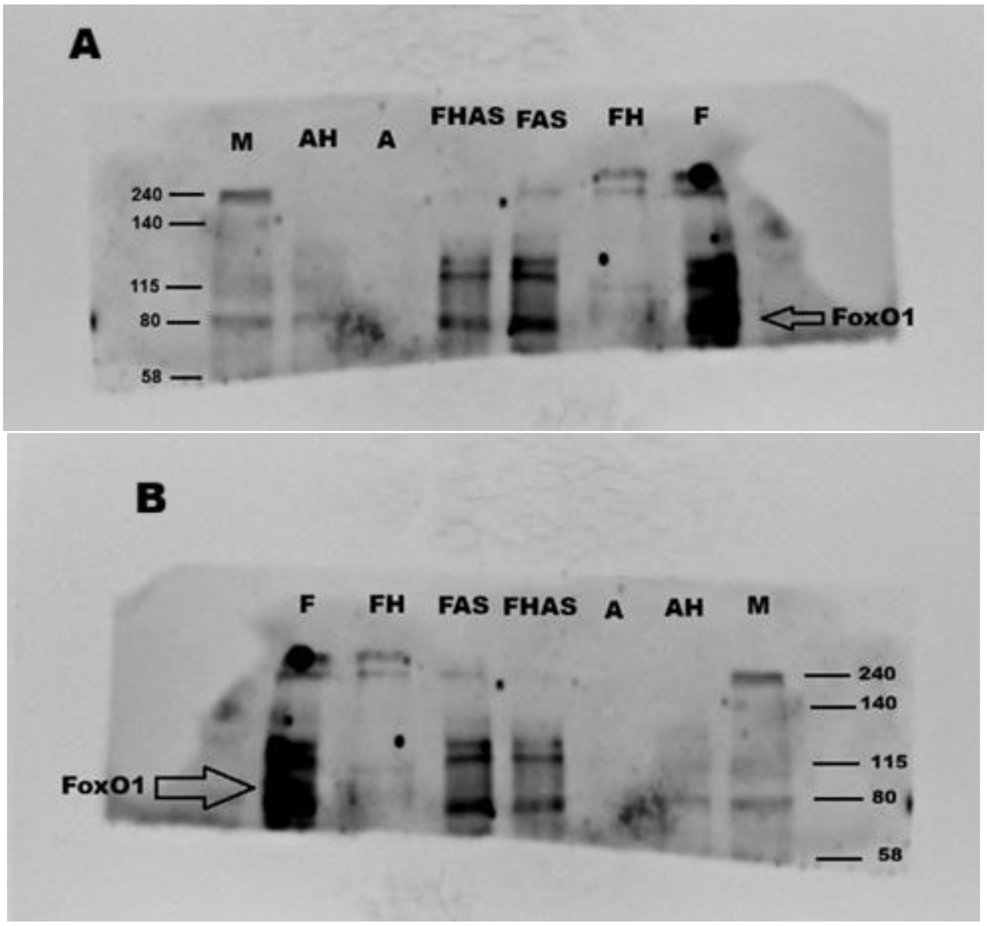

Figure S2: FoxO1 Western blot for Figure 3B. (A) Full scan (uncropped) original blot. (B) Horizontally flipped version corresponding to the orientation presented in the main manuscript. No analytical modifications were performed; all analyses were based on the original blot.

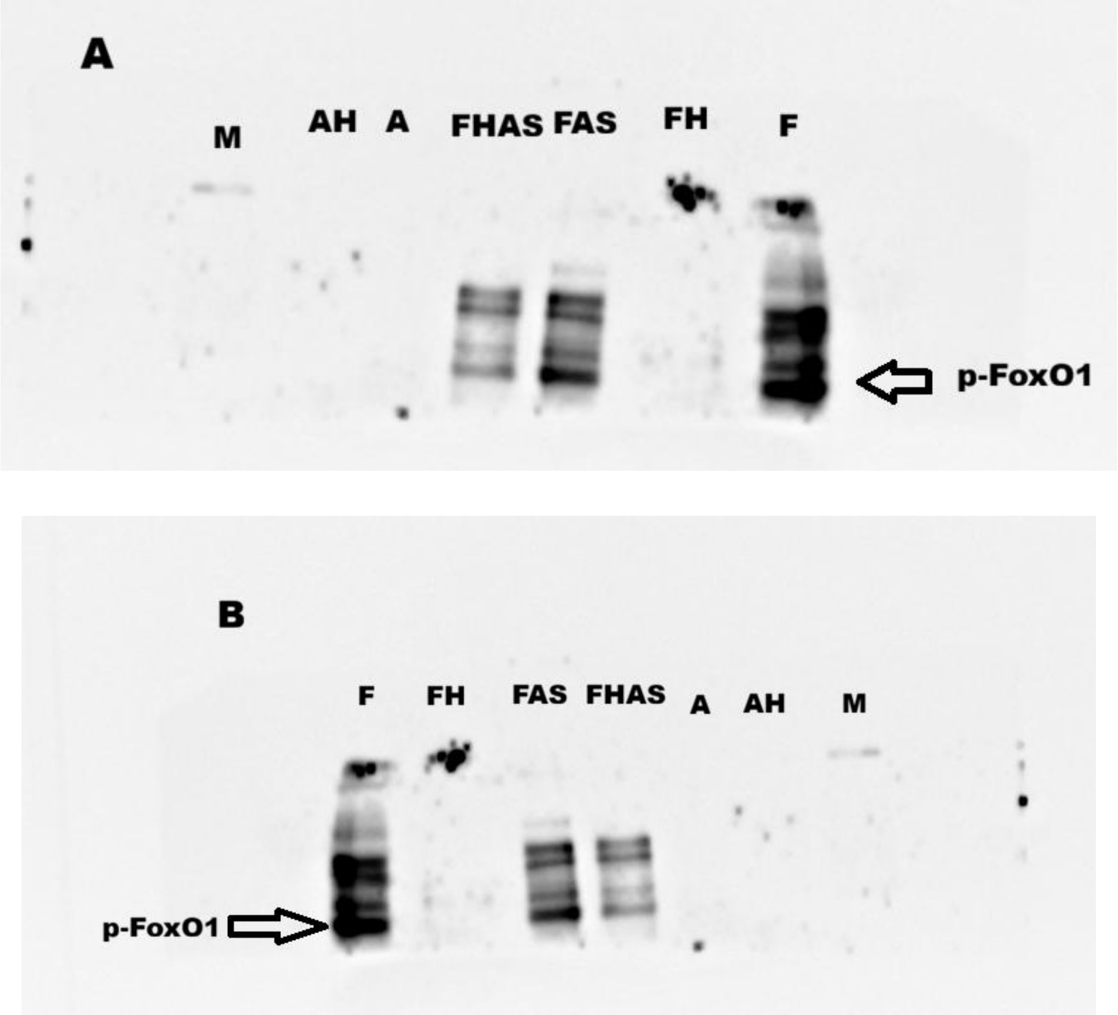

Figure S3: p-FoxO1 Western blot for Figure 3B. (A) Full scan (uncropped) original blot. (B) Horizontally flipped version corresponding to the orientation presented in the main manuscript. No analytical modifications were performed; all analyses were based on the original blot.

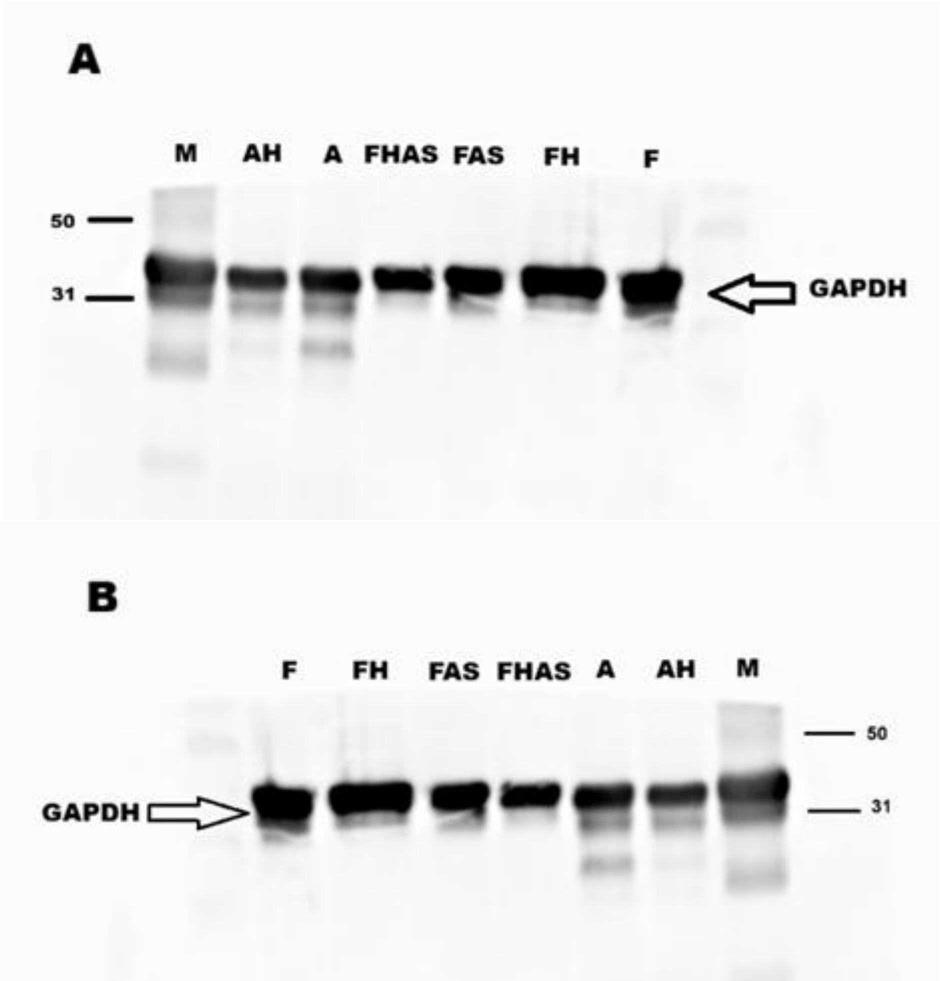

Figure S4: GAPDH Western blot for Figure 3B. (A) Full scan (uncropped) original blot. (B) Horizontally flipped version corresponding to the orientation presented in the main manuscript. No analytical modifications were performed; all analyses were based on the original blot.

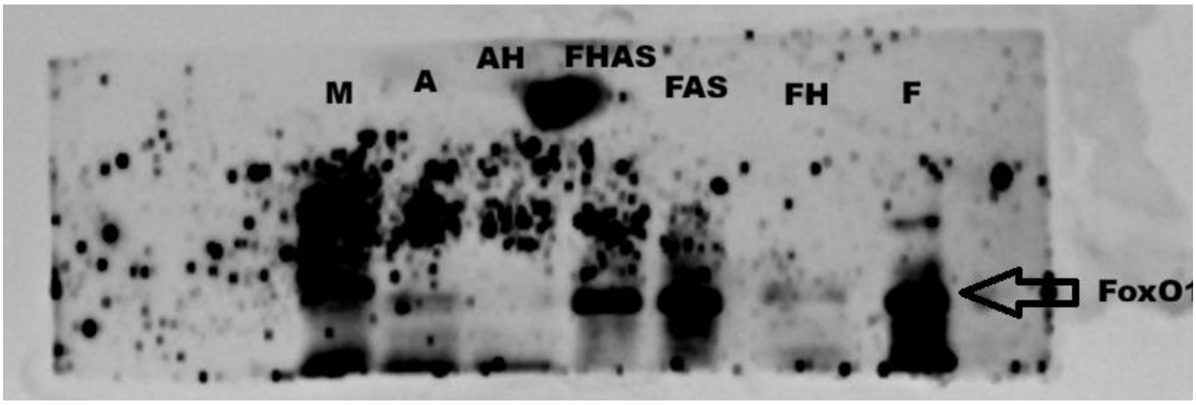

Figure S5: FoxO1 Western blot (second dataset). Full scan (uncropped) original blot. No flipped version is provided; all analyses were based on the original blot.

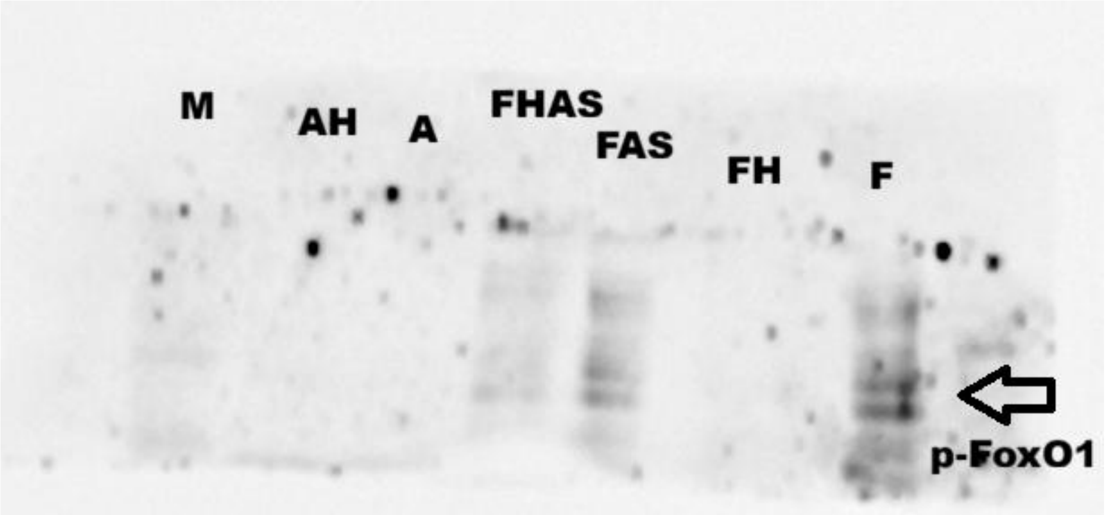

Figure S6: p-FoxO1 Western blot (second dataset). Full scan (uncropped) original blot. No flipped version is provided; all analyses were based on the original blot

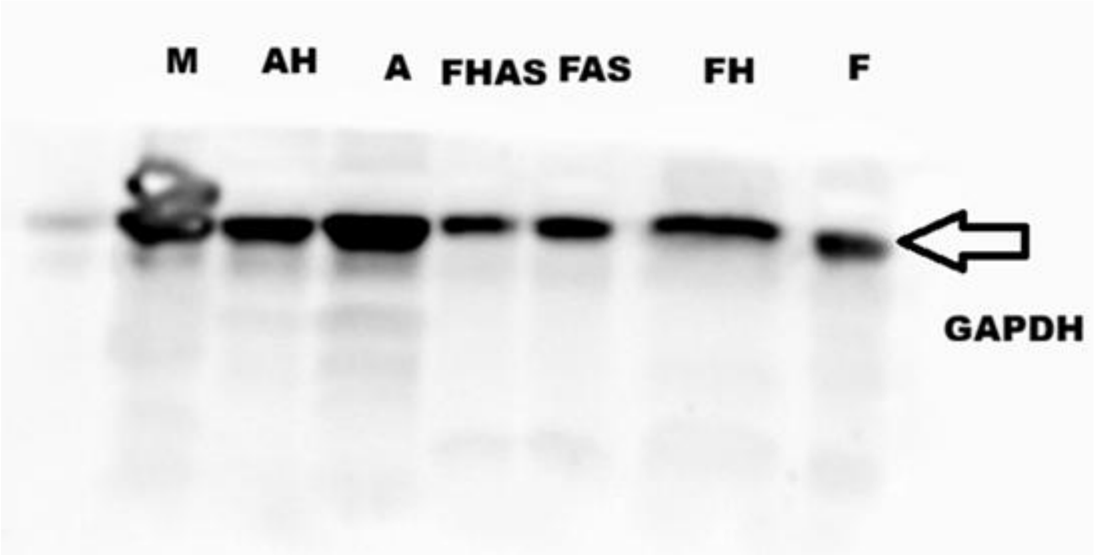

Figure S7: GAPDH Western blot (second dataset). Full scan (uncropped) original blot. No flipped version is provided; all analyses were based on the original blot.

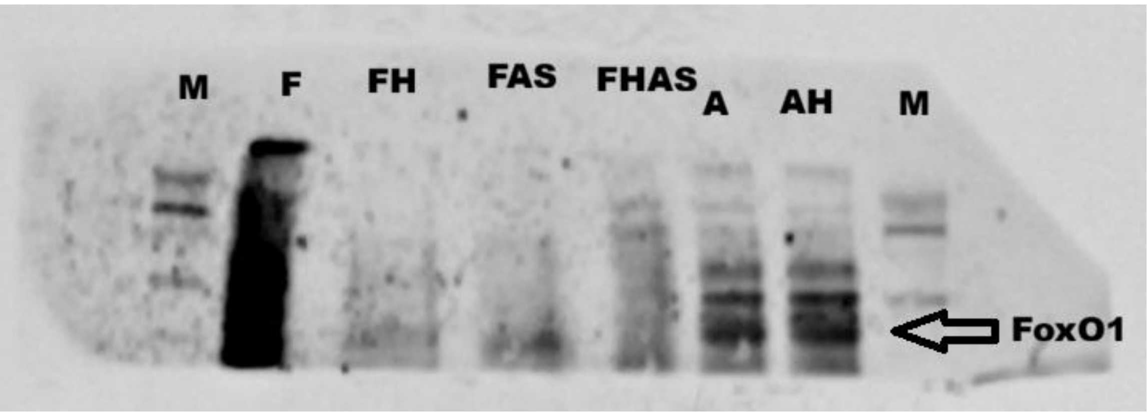

Figure S8: FoxO1 Western blot (third dataset). Full scan (uncropped) original blot. No flipped version is provided; all analyses were based on the original blot.

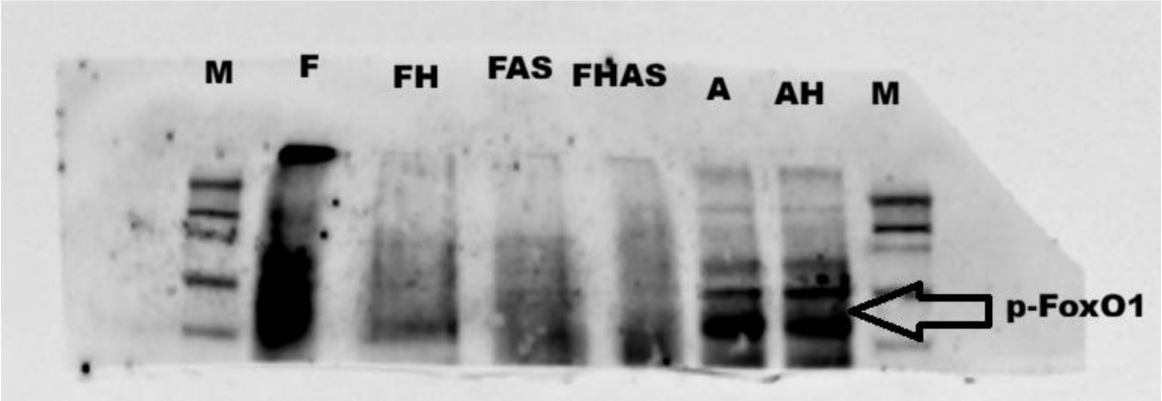

Figure S9: p-FoxO1 Western blot (third dataset). Full scan (uncropped) original blot. No flipped version is provided; all analyses were based on the original blot.

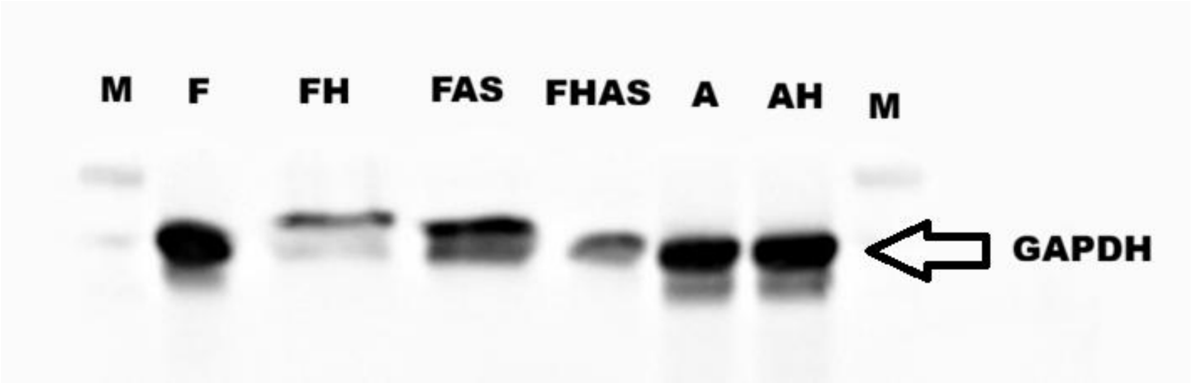

Figure S10: GAPDH Western blot (third dataset). Full scan (uncropped) original blot. No flipped version is provided; all analyses were based on the original blot.

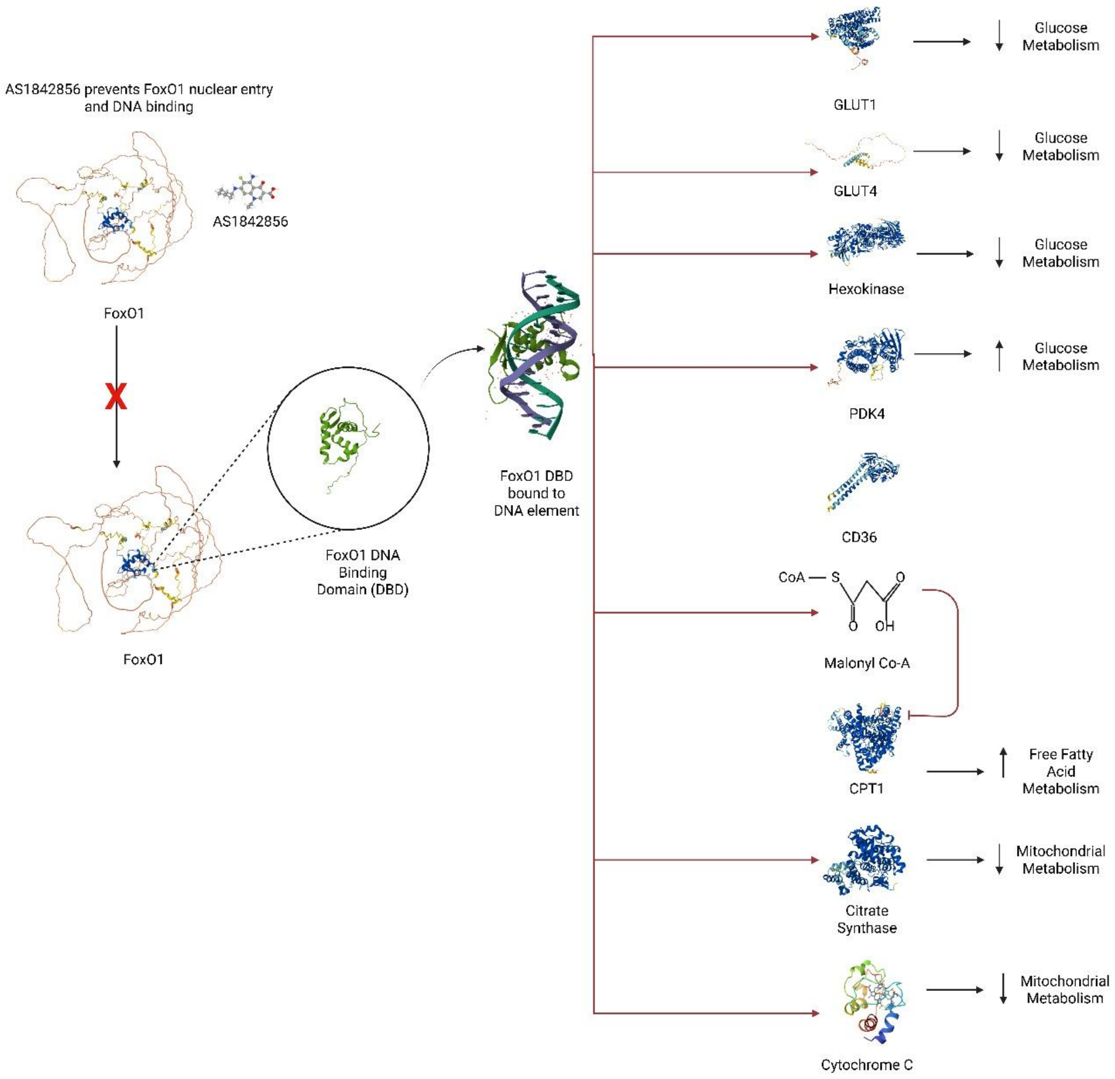

Figure S11: Structural model of FoxO1 DNA-binding domain interacting with a target DNA element. The small molecule AS1842856 prevents FoxO1 nuclear entry and DNA binding, thereby suppressing transcriptional regulation of downstream targets. As a result, glucose, free fatty acid, and mitochondrial metabolism are modulated. This schematic highlights the role of FoxO1 in orchestrating the metabolic shift in the fetal rat heart. Red arrows indicate transcriptional repression by FoxO1 or suppression of substrate production. Structural models were obtained from the AlphaFold database and the RCSB Protein Data Bank (PDB). Abbreviations: forkhead box protein O1 (FoxO1); DNA binding domain (DBD); glucose transporter 1/4 (GLUT1/4); pyruvate dehydrogenase kinase 4 (PDK4); cluster of differentiation 36 (CD36); carnitine palmitoyltransferase 1 (CPT1). Created in BioRender. william, W. (2026) https://BioRender.com/hp3wvfq, accessed on 16 March 2026.
